# Major hurdles to the use of tyrosine kinase inhibitors in clinical prevention/interception studies: Do preclinical studies with EGFR inhibitors suggest approaches to overcome some of the limitations

**DOI:** 10.3389/fcell.2023.1170444

**Published:** 2023-04-24

**Authors:** Konstantin H. Dragnev, Christo P. C. Dragnev, Ronald A. Lubet

**Affiliations:** ^1^ Dartmouth Cancer Center, Lebanon, NH, United States; ^2^ Colby College, Waterville, ME, United States; ^3^ Chemopreventive Agent Development Research Group, Division of Cancer Prevention, National Cancer Institute, Rockville, MD, United States

**Keywords:** tyrosine kinase inhibitor, cancer interception, EGFR inhibitiors, HER-2 inhibitors, clinical cancer prevention, cancer prevention trials

## Abstract

There are major hurdles to the use of tyrosine kinase inhibitors (TKIs) and any other agents with significant toxicities (which means practically the preponderance of potential effective agents) in the context of prevention/anti-progression (interception) studies. We will discuss epidermal growth factor receptor (EGFR) inhibitors as examples, both in a primary prevention setting, where agent(s) are administered to individuals with no cancer but who might be considered at higher risk due to a variety of factors, and in anti-progression/interception studies, where agent(s) are administered to persons with known preinvasive lesions (e.g., colon adenomas, lung nodules, ductal carcinoma *in situ* (DCIS), or pancreatic intraepithelial neoplasia (PanIN) lesions in the pancreas) in an attempt to reverse or inhibit progression of these lesions. Multiple potential hurdles will be examined, including: a) toxicity of agents, b) the likely range of subtypes of cancers affected by a given treatment (e.g., EGFR inhibitors against EGFR mutant lung adenocarcinomas), c) the availability of practical endpoints besides the blocking of cancer formation or pharmacokinetics related to the agents administered in a primary prevention study, and d) the interpretation of the regression or blockage of new preinvasive lesions in the anti-progression study. Such an anti-progression approach may help address some of the factors commented on regarding primary prevention (toxicity, potential target organ cancer subtypes) but still leaves major questions regarding interpretation of modulation of preinvasive endpoints when it may not be clear how frequently they progress to clinical cancer. Additionally, we address whether certain recent preclinical findings might be able to reduce the toxicities associated with these agents and perhaps even increase their potential efficacy. Antibodies and TKIs other than the EGFR inhibitors are not discussed because few if any had been tested as monotherapies in humans, making their efficacy harder to predict, and because a number have relatively rare but quite striking toxicities. Furthermore, most of the practical hurdles raised regarding the EGFR inhibitors are relevant to the other TKIs. Finally, we briefly discuss whether early detection employing blood or serum samples may allow identification of high-risk groups more amenable to agents with greater toxicity.

## 1 Introduction

**FIGURE 1:**
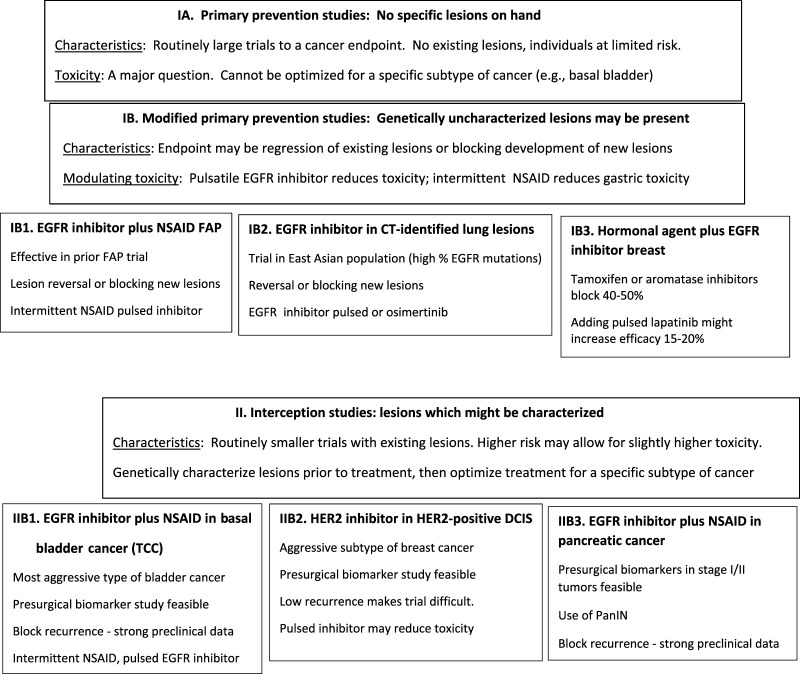
Examples of potential prevention and interception trials which include EGFR inhibitors.

The primary focus of this review is on some of the first generation EGFR inhibitors, e.g., gefitinib, erlotinib, lapatinib. This has been in contrast to certain of the third generation inhibitors, e.g., osmertinib, which preferentially interact with mutated forms of EGFR. This is predicated on the consideration that, as proposed for use in bladder, colon and pancreas, it appears that one is dealing with inhibition of wild type EGFR. Therefore, osmertinib which minimally effects wild type EGFR is unlikely to be highly effective. However, one could probably run a test for CT-scan identified lesions in an Asian population which has a very high incidence of EGFR mutant tumors with osmertinib since a high percentage of those lesions even without sequencing would be expected to have EGFR mutations. A systematic discussion of the widest range of small molecule EGFR inhibitors examining their specificity, target kinases, etc., has recently been published (Abourehab et al., 2021), with informative diagrams where EGFR inhibitors block various proteins. The additional anti-HER1/EGFR and anti-HER2 inhibitors that might be used are the antibodies, e.g., cetuximab, panitumumab and trastuzumab and their biosimilars. The difficulties with those agents are that they require intravenous administration, are likely to be quite expensive, and their combination with additional agents such as NSAIDs is not known. Examples of potential prevention and interception trials which include EGFR inhibitors that are discussed below, are on Figure 1.

## 2 Major hurdles to primary prevention studies

Primary prevention involves administering an agent(s) to a group of individuals with no cancer but who might be considered at higher risk for that type of cancer based on other known factors (e.g., age, hormonal status, known genetic factors) (Table 1).

**TABLE 1:** Trial characteristics and potential hurdles in primary prevention and interception studies using EGFR inhibitors.

		Primary prevention	Interception
Characteristics	Patient Population	Individuals with no known lesions, although they may be at higher risk due to genetic or other factors	Individuals with known lesions: Some lesions will not be characterized (multiple adenomas in FAP or CT-identified lung lesions). In other lesions (DCIS of the breast or transitional cell carcinomas (TCC) of the bladder) genomic characterization and identification of specific subtypes may allow a more tailored protocol
	Size of Trials	Typically greater than 1500 participants in the treatment and placebo arms	Typically trials of less 50 participants in each arm, may include one or more treatment groups, as well as placebo arm
	Primary endpoint	Prevention of cancer following 3–5 years trial	Tumor regression or blockage of new lesions—1–3 years endpoint. Modulation of biomarkers in defined lesions (DCIS or TCC) - ≤ 1 month
Potential hurdles	Toxicity	In a 3–5 years trial, only 1%–5% of individuals will develop a cancer. Most participants will have nothing to gain in the short run and significant toxicity will be unacceptable	Some syndromes (FAP) are in of themselves sufficiently deleterious that some treatment is necessary, and some toxicity will be acceptable. Others (PanIN) may not be at great risk of cancer in a few year window and any acceptable toxicity will be limited
	Cancer subtype	Unknown—prevalence of cancer in a given organ is roughly 3% during a lung cancer trial. If the EGFR inhibitor is only effective against EGFR mutant lung cancer and they represent less than 10% of the lung cancers in a cohort of smokers than 99% of the persons will have nothing to gain during the course of such a trial. This number gets even smaller if treatment is only 50% effective even in sensitive subtypes	May be known—FAP the precursor lesions will have multiple genetic signatures (all will have APC mutations, mixed KRAS status). DCIS and superficial bladder cancer will have a clear molecular lesion and signature will be available. Basal subtype of bladder cancer represents roughly 20% of bladder cancers but it has a worse prognosis than any of the other subtypes. Its susceptibility to EGFR inhibitors makes it a candidate for an intervention. Its poorer prognosis may allow for higher levels of toxicity which would be otherwise unacceptable
	Cost	If a drug is still on patent, it will not be sufficiently inexpensive to be used in the general public, even after a successful trial	The cost of the drug will be a smaller concern particularly if the lesions will result in insurance companies to reimbursing interventions for them
	Endpoints to employ in the trial	Development of new clinically detected cancers. This is a relatively hard endpoint in lung cancer, pancreatic cancer, colon cancer or breast cancer. It is less obvious in prostate where many or most cancers in an older population are unlikely to be the cause of death	Effects on biomarkers in the lesions, effects on reversing existing lesions, or blocking the formation new lesions. The relative risk of many of these preinvasive lesions (adenomas, PanIN, DCIS) to progress into clinical cancers is unclear. A clear biological interpretation what does regressing or reversing mean, as contrasted with statistical interpretation of these endpoints, may be difficult

### 2.1 Hurdle 1: toxicity as a primary hurdle to primary prevention studies

A primary obstacle to long-term clinical primary chemoprevention studies and acceptance is toxicity. For example, tamoxifen and the aromatase inhibitors have proven highly effective in the prevention of estrogen receptor-positive (ER+) breast cancer in large-scale clinical trials (Fisher et al., 1998; Goss et al., 2011). In the prevention trials, these agents were tested at the same doses employed in the therapy of estrogen/progesterone receptor-positive (ER + PR+) tumors. Non-etheless, it has been difficult for at-risk women to take these agents. In the case of tamoxifen, a major hurdle is the increase in endometrial cancer associated with treatment. In the case of the aromatase inhibitors, drawbacks include postmenopausal symptoms, muscle and joint pain, and osteoporosis. Another major class of agents that has shown therapeutic efficacy but has minimally advanced to a prevention setting are the tyrosine kinase inhibitors (TKIs). Specifically, epidermal growth factor receptor (EGFR) inhibitors are clearly effective clinically in the treatment of EGFR mutant lung cancer (Ramalingam et al., 2020), and HER2 inhibitors are active in HER2-positive breast cancer (Cortes et al., 2022) (Park et al., 2009). Furthermore, the EGFR inhibitor erlotinib proved active when combined with the NSAID sulindac in a polyp trial with familial adenomatous polyposis (FAP) patients (Samadder et al., 2016; Samadder et al., 2018). However, these agents at their recommended daily dosing cause significant and common toxicities, such as acneiform rash and diarrhea (Guttman-Yassky et al., 2010; Shepherd et al., 2005; Burstein et al., 2008; Yang et al., 2012), which have been a barrier to their routine use in a prevention setting. Preclinical prevention studies (see below) have raised the possibility of reducing such toxicities by employing intermittent dosing, weekly dosing, or a combination of agents. If validated in clinical trials, these methods could be key to improving the relative efficacy to toxicity index and increase the potential use of these inhibitors in prevention/anti-progressions studies.

### 2.2 Hurdle 2: what is the likely subset of a given form of cancer that will be prevented by an agent: are preclinical data sufficient for a small phase II?

In the case of tamoxifen and the aromatase inhibitors, one had clear data from large adjuvant trials that these agents used as monotherapies would prevent the development of the preponderance of ER + PR + HER2-negative breast cancers (Luminal A) (Perou et al., 2000). This was based on their ability to affect breast cancer recurrence and/or metastasis in the adjuvant setting (Howell et al., 2005). However, there was a second parameter observed in the large adjuvant trials with tamoxifen and the aromatase inhibitors that closely paralleled prevention trials, specifically the ability of these agents to prevent the development of breast cancer in the contralateral breast (Goss et al., 2005; Ruhstaller et al., 2019). These contralateral tumors are thought to represent newly arising tumors in contrast with recurrences or metastatic cancers. These studies showed that tamoxifen was relatively effective in the prevention of contralateral breast cancers (60%), while the aromatase inhibitors were very effective in preventing >80% of contralateral cancers. The prevention trials reproduced these results with tamoxifen, reducing the incidence of ER + breast cancer 35%–55% in various trials, while the aromatase inhibitors decreased ER + tumors by almost two-thirds. ER + breast cancers represent 70%–80% of all breast cancers in most studies. Thus, if an agent inhibited ER + tumors by 75% in a large prevention trial of breast cancer, it would be expected to inhibit 50%–60% of all breast cancers. This is a reasonable approximation of what was seen in the two large prevention trials (Fisher et al., 1998; Goss et al., 2011). Furthermore, neither tamoxifen nor the aromatase inhibitors appeared to affect the expected numbers of ER-negative breast cancers relative to placebo, implying you are not shunting or specifically augmenting the development of ER-negative tumors. In contrast, adjuvant results with agents in most other forms of cancer (lung, colon, pancreas, bladder) do not yield any similar contralateral type “prevention” results, so one must try to extrapolate from results in an adjuvant setting that uses endpoints such as recurrence free survival (RFS) or overall survival (OS), which may seem less relevant for prevention. However, a second problem is that with the exception of treating EGFR mutant lung cancers, the majority of examples of the use of EGFR inhibitors, and most other TKIs have been in combination with a second agent, most typically a standard cytotoxic agent (Geyer et al., 2006). However, there appears to be sufficient data in HER2 tumors to propose that HER2 small molecule inhibitors by themselves will be effective. Therefore, it is more difficult to determine whether a given agent will be functional on its own. This leads to the question whether positive results in a preclinical setting or effective preclinical results with EGFR inhibitors alone or in combination with other known preventive agents would be sufficient to initiate at least a small phase II prevention/anti-progression trial. One could certainly hope that preclinical data in a relevant animal model with or without clinical data on a compound or a preclinical combination with a second standard agent would be sufficient to perform a small presurgical neoadjuvant trial which might support a prevention trial.

These specific examples of EGFR inhibitors or HER2 inhibitors exemplify the problem for TKIs, or probably most targeted therapies in a primary prevention setting. EGFR mutant lung adenocarcinomas in a Caucasian population represent <15% of all adenocarcinomas. Since adenocarcinomas represent at most 50% of all lung cancers worldwide, EGFR mutant lung adenocarcinomas will represent <7% of all lung cancers. Similarly, an HER2 inhibitor by itself is likely to affect only the roughly 20% of breast cancers that overexpress HER2 and are either ER positive or ER negative. Thus, it would not be the basis for a primary prevention trial, although it might significantly improve a trial in conjunction with an anti-hormonal agent (Fisher et al., 1998; Goss et al., 2011).

### 2.3 Hurdle 3: question of treatment duration following a general prevention trial

In the metastatic setting, one routinely expects to continue treatment until the tumor progresses or until you have a striking pathologic response. In the adjuvant setting, as in the case of tamoxifen and the aromatase inhibitors, it was demonstrated that 5 years of treatment is roughly as good as 10 years. However, the prevention trial with the aromatase inhibitor anastrozole raises the question of how long to treat (Cuzick, 2003; Cuzick et al., 2014; Forbes et al., 2016; Cuzick et al., 2020). This recent trial showed that while total breast cancers were reduced by roughly 50% during the first 5 years when the agent was administered, this dropped to roughly 30% efficacy during the next 5 years during which anastrozole was withdrawn. In a phase I or phase II prevention trial, the endpoints are likely to be biomarkers of efficacy and/or blocking some preinvasive lesion. In a large prevention trial, the question of duration of treatment is a major question. You do not have a progressing lesion. You can look for the development of new cancers and that would presumably be the endpoint for any large-scale trial (Fisher et al., 1998; Goss et al., 2011). Thus, optimistically, the incidence of the cancer of interest is reduced by 50%. That is likely to be sufficient to yield a statistically positive trial. However, do you have to keep administering the agent chronically? Only 2%–3% of persons will develop a tumor in a large primary prevention trial (Fisher et al., 1998; Goss et al., 2011). Do you keep the 97%–98% of the group who have not developed a cancer on the agent? This is a significant question. Furthermore, particularly for the TKIs, the possibility of developing resistance is always an open question. Quite obviously for the TKIs, this is perhaps the major question in therapy trials where one has a tumor which is likely to keep turning out new mutated variants. However, from limited highly effective prevention trials with tamoxifen or aromatase inhibitors in breast or NSAIDs in colon, it does not appear that you are getting resistant tumors readily. The rationale for this lack of resistance may be that you do not have a significant number of cells from more advanced lesions which are more likely to undergo mutations. However, there is certainly a question of whether these results with hormonal agents and NSAIDs are relevant for the TKIs.

### 2.4 Hurdle 4: cost, who is paying?

The cost of a large-scale clinical prevention trial is quite substantial. However, if the result obtained is relatively striking then it may be more than worth the cost. However, if the agent employed is still on patent, then the cost is likely to prove prohibitive for general use in a population. Quite obviously, if an agent has significant toxicity and may require observation by medical practitioners due to toxicity, this likely makes it untenable for primary prevention.

## 3 Interception (anti-progression) studies: TKI use in individuals with preexisting lesions and examples of such populations

Although primary prevention is often what people think of when they discuss prevention as listed above, the use of such an approach is complex in general and potentially fraught for a class of agents such as the TKI inhibitors (using as an example the EGFR inhibitors) (Table 1). The existence of populations with precursor lesions partially overcomes certain of the problems associated with pure primary prevention trials: 1) The mere presence of a lesion: If a lesion is available which helps to define the subtype of cancer, then one may be able to use a specific class of agents more rationally. 2) Precursor lesions and potential endpoints: If there are specific precursor lesions, either biomarker alterations in the lesion or lesions that one can measure, or regression of the lesion or blockage of development of new lesions, one may be able to define endpoints for a phase I/II trial. 3) Persons with precursor lesions must be considered at higher risk: Presumably, the existence of the lesion puts the individual at greater risk of developing the specific caner and therefore makes somewhat higher levels of toxicity more acceptable. 4) Precursor lesions may make some treatment necessary: Certain precursor lesions (e.g., multiple polyps associated with FAP) virtually require some treatment. Furthermore, precursor lesions such as ductal carcinoma *in situ* (DCIS) in the breast or transitional cell carcinomas (TCCs) in the bladder are routinely treated with surgery and some adjuvant treatment.

### 3.1 Examples of precursor lesions which might define individuals for interception (anti-progression) trials

#### 3.1.1 Persons with lung precursor lesions determined by CT scanning

(Table 1) CT scanning of lungs, although far from foolproof, may help identify persons with early lesions (nodules) in the lung (National Lung Screening Trial Research et al., 2011). These and other techniques are felt to determine lesions which may be the precursors to primarily lung adenocarcinomas (Hammer and Byrne, 2022). Regarding the use of EGFR inhibitors, such an approach would be of limited use regarding potential prevention trials, particularly in Caucasian smokers, since few of these lung adenocarcinomas have EGFR mutations. However, if such a study were performed in an East Asian population of smokers or non-smokers, then the prevalence of EGFR mutations (Shi et al., 2014) is likely to be sufficient to justify such a trial, particularly if one might enhance the efficacy of these inhibitors and potentially partially reduce their toxicity (see below). There is a rapidly evolving field of risk assessment tools in addition to CT screening for inclusion in lung cancer prevention/interception studies (Tammemagi et al., 2013; Mathios et al., 2021).

#### 3.1.2 Persons with FAP (Familial adenomatous polyposis)

Due to germline mutations in APC (adenomatous polyposis coli), individuals with FAP have literally scores to hundreds of adenomatous polyps. Because of the presence of a great number of lesions and the expectation that one or more of these lesions will progress to invasive colon cancer, some clinical intervention appears necessary. There was a positive FAP trial combining the EGFR inhibitor erlotinib together with a relatively low dose of the NSAID sulindac which resulted in a fairly striking clinical effect (Samadder et al., 2016; Samadder et al., 2018). This small trial was based on strong preclinical data showing that the combination of an EGFR inhibitor and an NSAID was profoundly effective in animal models of FAP (Buchanan et al., 2007). This is in contrast to adjuvant clinical data which show that anti-EGFR antibodies, but not EGFR small molecule inhibitors, are effective in colon cancer (Taieb et al., 2014). This raises the question whether these data reflect a greater sensitivity of the preinvasive lesions or whether a small molecule EGFR inhibitor specifically combined with an NSAID might be effective even in an adjuvant setting.

#### 3.1.3 Treatment/interception trials for pre-invasive DCIS lesions

DCIS are precursor pre-invasive lesions that are frequently identified at screening mammography or during breast cancer surgery. These are commonly treated with surgery, often followed by adjuvant radiation and hormonal therapy. While this is considered therapy by most oncologists, the objective is to prevent the development of invasive cancer and could serve as an interception (anti-progression) model. For DCIS lesions overexpressing HER2, trastuzumab has demonstrated activity in clinical trials (Cobleigh et al., 2021; von Minckwitz et al., 2012). A trial of the small molecule HER2 inhibitor lapatinib in DCIS was terminated early for low accrual, despite strong preclinical evidence for activity (Farnie et al., 2014).

#### 3.1.4 Pancreatic intraepithelial neoplasia (PanIN)

PanIN is a histologically well-defined precursor to invasive ductal adenocarcinoma of the pancreas. PanINs are relatively common lesions, particularly in an elderly population. These lesions typically have a KRAS mutation and often alterations in P16 as well. These are the two most common genomic alterations observed in invasive ductal adenocarcinomas of the pancreas. However, it is still not clear what percentage of PanIN lesions progress to pancreatic adenocarcinoma (Delpu et al., 2011).

#### 3.1.5 Basal subtype of transitional cell carcinoma (TCC) of the bladder

TCC is the most frequently observed tumor in the bladder. TCC arises from the cells lining the bladder and is routinely diagnosed during the pre-invasive stage. At the molecular level, there are 5 major subtypes of bladder cancer, including luminal (HER2/3 high, papillary) (Cancer Genome Atlas Research, 2014), HER2-expressing, and basal (squamous cell, mesenchymal cell) (Hedegaard et al., 2016; Choi et al., 2014; Kamoun et al., 2020). The basal cell cancers, which represent roughly 20%–25% of bladder cancers, appear to be responsive to EGFR inhibitors. Similarly, the HER2-overexpressing category, which has a far better prognosis than the basal, might be a candidate for treatment with HER2 inhibitors. Lapatinib, a combined EGFR inhibitor and HER2 inhibitor, when combined with the NSAID piroxicam, profoundly increased progression free survival and overall survival in dogs with invasive bladder cancers (Maeda et al., 2022).

#### 3.1.6 Head and neck oral premalignant lesions and squamous cell cancer of the head and neck

Carcinogenesis of the head and neck area provides a classical example of the multistep process leading to invasive malignancy. With easily observable oral premalignant lesions that could undergo serial biopsies, several trials assessed the activity of EGFR TKIs alone or in combinations with other agents for prevention of invasive cancer, or evaluating surrogate biomarkers in patients with oral premalignant lesions. The combination of erlotinib and celecoxib led to 63% histologic response rate that correlated with EGFR pathway inhibition (Saba et al., 2014). Green tea polyphenon E and erlotinib resulted in 47% complete pathologic response with an excellent 66.3% 5-year cancer-free survival. Phosphorylated ERK was correlated with response to treatment, among the biomarkers that were studied. (Shin et al., 2020). However, a randomized trial with erlotinib vs. placebo did not show improvement in the primary endpoint of cancer-free survival (William et al., 2016). Preclinical data suggest that MET activation may be present as an early driver in premalignant lesions and could become a target for chemoprevention of oral cancer (Saintigny et al., 2018). A proof of concept phase II clinical trial of metformin to target PI3K/mTOR signaling for patients with oral premalignant lesions showed modest clinical responses and decreased mTOR activity correlating with the histological and clinical responses. The observed significant modulation of the PI3K/mTOR pathway indicated that trials with other PI3K inhibitors are warranted (Gutkind et al., 2021).

## 4 Possible ways to reduce the toxicities associated with EGFR inhibitors to facilitate their use in prevention/interception studies

Preclinical prevention studies have raised the possibility of reducing toxicity by employing intermittent dosing, weekly dosing, lower dosing, or a combination of agents (Padda et al., 2013) (Table2). Once validated in clinical trials, these methods could be a key to improving the therapeutic index of preventive/anti-progression (interception) agents and thus their public acceptance.

**TABLE 2:** Clinical interception trials which might be performed: the rationale for such trials, potential trial endpoints and altered dosing which might reduce toxicity and/or increase efficacy (See also Figure 1).

Target	FAP	EGFR mutant lung lesions	PanIN lesions	Basal subtype of bladder transitional cell carcinoma	HER2 -expressing DCIS
Clinical Trial	EGFR inhibitor and an NSAID	EGFR inhibitor in CT-identified lung lesions in Asian population	EGFR inhibitor and an NSAID	EGFR inhibitor and an NSAID	A HER2 inhibitor
Rationale	Profound efficacy of combining COX 2/1 inhibitors with erlotinib preclinically, leading to a successful clinical trial combining a standard dose of erlotinib and low dose of sulindac. The individual arms were not tested	EGFR inhibitors are highly effective even against EGFR mutant lung cancers. A high percentage of CT- identified lesions in an Asian population will be EGFR mutant	Individual efficacy of both COX 2/1 inhibitors and EGFR inhibitors in pancreatic models preclinically. The combination has not been used preclinically, that is required. This combination of agents was effective in FAP giving both relevant toxicity data and the hope that these combinations may translate clinically based on preclinical data	Poor prognosis of this specific type of TCC. Preclincally profound efficacy of combining COX 2/1 inhibitors with EGFR inhibitors. Limited human data of efficacy of EGFR inhibitors in this subtype and at least some epidemiologic data implying efficacy of NSAIDs in reducing bladder cancers. Epidemiologic studies did not test for bladder cancer subtypes	Trastuzumab is effective both in invasive and non-invasive HER2 overexpressing lesions. There are clinical data in later stages such that it is likely to be effective early
Endpoints	Regression of existing polyps and/or prevention of new polyps	Regression of existing lesions and/or inhibition of EGFR mutant cancers, depending on how frequently these progress	Regression of existing lesions and/or prevention of new lesions. Interpretation of such a study limited by the natural history of these lesions	Blocking recurrence and/or progression of lesions	Biomarker modulation in lesions (almost certain to work) and/or progression of lesions (relatively infrequent)
Altered dosing	Combine weekly EGFR inhibitor (may reduce toxicity) with intermittent NSAID dosing (reduce gastric toxicity but keep high efficacy)	Weekly EGFR inhibitor which may reduce toxicity and might increase efficacy (does so in preclinical studies)	Combine weekly EGFR inhibitor (which may reduce toxicity) with intermittent NSAID dosing which should reduce gastric toxicity. Based on preclinical data the combination could be highly effective	The combination of weekly EGFR inhibitor (which may reduce toxicity) with intermittent NSAID dosing is highly effective preclinically	The use of weekly HER2 inhibitor may reduce toxicity and increase efficacy

A problem with targeted therapies developed for cancer treatment, such as the EGFR inhibitors erlotinib, gefitinib, and lapatinib, is that they all can cause acneiform rash and diarrhea when given daily. Studies in humans treating brain metastases from either EGFR mutant lung cancers or HER2-overexpressing breast cancers demonstrated that pulsatile weekly dosing with erlotinib or lapatinib, at up to 7-fold the standard daily dose, was effective and paradoxically caused less toxicity than daily dosing. This high dose pulsatile dosing was initiated to deal with brain metastases from EGFR mutant lung cancers and HER2-overexpressing breast cancers. Daily dosing of these agents did not reach effective levels in the brain following standard daily dosing, presumably due to the blood brain barrier. Therefore, weekly pulsatile dosing was employed on the rationale that one would achieve higher levels in the brain. It appears that the approach has been relatively effective in numerous human trials (Milton et al., 2006; Grommes et al., 2011; Morikawa et al., 2019). In preclinical studies, weekly dosing with a number of EGFR inhibitors, including erlotinib and gefitinib, EGFR inhibitors were equally effective as daily dosing in breast, bladder and colon cancer models, and potentially more effective in two lung cancer models (Lubet et al., 2013; Zhang et al., 2017; Mohammed et al., 2020; Ulusan et al., 2021). A particularly striking example of the efficacy of EGFR inhibitors combined with an NSAID has been recently reported in dogs (Maeda et al., 2022). The combination of the HER inhibitor lapatinib (which inhibits both HER1/EGFR and HER2) increased progression free survival in dogs with invasive bladder tumors almost 2-fold, and increased overall survival roughly 2-fold as well. The comparator arm for these trials was piroxicam alone, which is a fairly standard way to treat these tumors. These results similarly appear more effective than standard chemotherapies (e.g., mitoxane). However, there was not a direct comparator arm in this study. These results raise the question whether this type of approach might be relevant to human bladder cancers as well. These studies did not test altered dosing, which we have recently examined. However, our observation of efficacy with multiple models in rats and mice and the implication that it will at a minimum reduce toxicity and might increase efficacy warrants consideration. As a pure aside, most of our early studies in bladder used erlotinib and gefitinib because our model paralleled human basal bladder cancer and overexpressed EGFR but not HER2. However, HER2 is overexpressed in many other human and canine subtypes of bladder cancer and may be relevant to the use of this approach for a wide variety of bladder tumors (preinvasive and invasive) in humans. Parenthetically, as is to be expected, lapatinib, an EGFR and HER2 inhibitor, was similarly effective when given alone or in combination with an NSAID in our basal bladder cancer model (Lubet et al., 2021). Examples of the efficacy of this approach are shown in Table 2. A number of papers have shown reduced toxicity with weekly dosing in animal models, although the skin lesions examined are not exactly the same as acneiform rash in humans (Zhang et al., 2017; Ulusan et al., 2021). Also, the combination of weekly dosing of an EGFR inhibitor together with an NSAID has looked striking in preclinical models of colon and bladder cancer.

Potential trials employing weekly EGFR inhibitor administration (Table 2):

1. Weekly dosing with an EGFR inhibitor in an Asian population (that has a high incidence of EGFR mutant lung cancers) with pulmonary nodules determined by CAT scans (Shi et al., 2014)
2. Weekly dosing of an EGFR inhibitor together with an NSAID (see below) in individuals with a) FAP, b) pancreatic precursor lesions (PanINs), and c) basal TCC of the bladder (Mohammed et al., 2020)
3. Potential trial of lapatinib in DCIS. HER2 overexpression is often observed in DCIS in the breast and appears to be associated with a higher incidence of recurrence and progression to invasive breast cancer. This is routinely treated with surgery plus radiation. Trastuzumab can be employed in this setting with at least hints of efficacy, but it requires intravenous administration.

## 5 Enhanced efficacy and potential toxicities associated with combinations of agents

There are both preclinical and some clinical data that combinations of certain agents may yield strong efficacy. However, any potential toxicity problems that may arise may have to be determined clinically. Common toxicities, such as acneiform rash or diarrhea for EGFR inhibitors or gastric bleeding for NSAIDs, could be determined in a limited Phase I trial.

Two of the more successful Phase II clinical prevention trials that involved combinations of agents were performed in colon and were based on preclinical data. In the first, the combination of an NSAID (piroxicam) and DFMO was shown to be highly effective preclinically in a colon model almost 30 years ago (Reddy et al., 1990). This led to a colon adenoma prevention trial combining the NSAID sulindac and DFMO, which prevented adenomas by roughly 60% (Meyskens et al., 2008). In the second, which is immediately relevant to the present paper, the EGFR inhibitor erlotinib administered with sulindac was highly effective in persons with FAP (Samadder et al., 2016; Samadder et al., 2018). The efficacy of this combination was first observed in Min mice by DuBois and coworkers (Buchanan et al., 2007) and most importantly, was confirmed in a human trial of FAP which looked at the regression of preexisting polyps. As mentioned above, the toxicities observed were a combination of those seen with the individual agents. Regrettably, for each of these trials, which were relatively small phase II trials, neither of the individual agents were administered concurrently as comparator arms. This makes it more difficult to determine whether the combination of agents was more or less toxic than the sum of the individual agents. In the case of the NSAIDs, this is particularly relevant since they were administered at less than their standard doses.

Potential trials employing EGFR inhibitor plus an NSAID:

1. Repeat of FAP Study: The prior FAP study examining erlotinib plus an NSAID can be performed using a weekly dose of erlotinib. This is likely to be both highly effective and plausibly less toxic than the standard dosing. The combination of pulsatile EGFR inhibitor and intermittent NSAID was found to be profoundly effective in a rat model of FAP (Ulusan et al., 2021).
2. Interception (anti-progression study) on pre-invasive pancreatic lesions (e.g., PanIN lesions): The combination of an EGFR inhibitor and a Cox 1/2 inhibitor is highly effective preclinically (Rao et al., 2015).
3. Interception (anti-progression) study in basal subtype of bladder cancer: Erlotinib plus an NSAID can be administered using a weekly dose of erlotinib. This is likely to be both more effective and plausibly less toxic. The combination of pulsatile EGFR inhibitor and intermittent NSAID was found to be profoundly effective in a rat model of basal bladder cancer (Mohammed et al., 2020). Perhaps even more surprisingly the combination of lapatinib plus piroxicam was highly effective against all bladder cancers in dogs (Maeda et al., 2022).

## 6 Is there a potential solution for the hurdles raised in primary prevention and interception trials?

In science in general and oncology in particular, significant advances, at least conceptually, often come out of some technical advance. The potential technical advance that is starting to come into clearer focus is the possibility of early detection of incipient tumors in blood or some other body fluid (Liu et al., 2020; Mathios et al., 2021). By definition, the signal will reflect some existing lesion that has to be of a sufficient size to give a significant signal in any relevant assay. The signal would identify both a type of cancer and hopefully some subtype of cancer (e.g., EGFR mutant lung cancer or basal bladder cancer) but would not offer a complete genome to sequence. At the time of diagnosis, it might not offer a clear lesion to examine. Would administering agents to such a lesion constitute prevention or is it a therapy? That may be somewhat of a semantic argument. This yields a number of advantages in terms of a patient population. First, it offers a population that is likely to advance to frank clinical cancer. Second, the biomarkers that allowed for the identification of patients with incipient cancers are likely to be useful in monitoring the efficacy of any treatments. Third, the incipient lesions are more advanced and therefore require more effective agents (e.g., TKIs). This greater likelihood of progressing to clinical cancer would appear to make this group of patients more receptive to treatments with some but easily manageable toxicities. Progressing into this population will hopefully allow the use of TKIs in certain of these populations, particularly if some of the preclinical methods mentioned above do reduce the toxicity of some of the TKIs.

## 7 Bottom line

A. As outlined above, there are multiple hurdles to any potential use of EGFR inhibitors in particular or any other TKIs generally in a primary prevention trial. These include the fact that a potential subtype of sensitive tumors (EGFR mutant lung cancer or HER2-amplified breast cancer) to target in such a blind primary prevention trial is unclear. Furthermore, there are too many toxicities associated with daily dosing to make it viable unless some of the newer TKIs have more limited toxicity.
B. In an interception trial, it would seem that one can determine subtypes of cancers of particular organs (EGFR mutant lung adenocarcinomas) or precursor lesions (HER2-overexpressing DCIS) that might be susceptible to an EGFR receptor inhibitor. There are still questions about potential endpoints to employ. However, the presence of lesions raises the possibility of examining alterations in lesions by various -omic methodologies. Furthermore, if someone has existing lesions, some increase in toxicity will be more acceptable.
C. There are alterations in dosing and potential combinations of agents which might make the use of TKIs more acceptable. These were discussed above. It appears that pulsatile dosing of TKI inhibitors may prove useful in certain subtypes of cancer. Furthermore, the combination of an EGFR inhibitor plus an NSAID (a known preventive agent) appears particularly promising in anti-progression studies such as FAP.
